# Self-Efficacy of Family Caregivers of Older Adults With Cognitive Impairment: A Concept Analysis

**DOI:** 10.1093/geroni/igaa057.246

**Published:** 2020-12-16

**Authors:** Tarik Khan, Karen Hirschman, Matthew D McHugh, Mary Naylor, Tarik S Khan

**Affiliations:** 1 University of Pennsylvania, PHILADELPHIA, Pennsylvania, United States; 2 NewCourtland Center for Transitions and Health, Philadelphia, Pennsylvania, United States; 3 University of Pennsylvania, Philadelphia, Pennsylvania, United States

## Abstract

The purpose of this concept analysis is to address fundamental gaps in the understanding of self-efficacy in family caregivers of older adults with cognitive impairment, including updating the 26-year-old concept analysis with a contemporary definition. With the first of the baby boomers set to turn 75 in 2021, the growing number of Americans with Alzheimer’s disease is predicted to more than double over the next 30 years, while the pool of potential family caregivers is likely to diminish by half. Research demonstrates that increased self-efficacy can help family caregivers of older adults with Alzheimer’s and other types of cognitive impairment experience lower burden and depressive symptom severity. This study utilized Walker and Avant’s method of concept analysis, an eight-step iterative process that helps to clarify the meaning of ambiguous concepts. A literature review was conducted from July 1993 through March 2019 using PubMed/MEDLINE, Scopus, CINAHL, and Embase. Eight defining attributes of this concept were identified. The revised definition of self-efficacy in this population is “a family caregiver’s confidence in their ability to: manage behaviors and other caregiving stresses, control upsetting thoughts, acquire medical information, manage medical issues, obtain self-care, access community supports, assist with activities of daily living and other care, and maintain a good relationship with a relative, friend, or neighbor of an older adult with cognitive impairment.” Practice implications include tailoring interventions to improve family caregiver self-efficacy. Policy implications include fostering evidence-based health strategies through payment and delivery incentives that further support caregiver self-efficacy.

